# Multifaceted Population Structure and Reproductive Strategy in *Leishmania donovani* Complex in One Sudanese Village

**DOI:** 10.1371/journal.pntd.0001448

**Published:** 2011-12-20

**Authors:** Virginie Rougeron, Thierry De Meeûs, Mallorie Hide, Georges Le Falher, Bruno Bucheton, Jacques Dereure, Sayda H. El-Safi, Alain Dessein, Anne-Laure Bañuls

**Affiliations:** 1 Laboratoire MIVEGEC (UMR IRD 224-CNRS 5290-Université Montpellier 1), Montpellier, France; 2 UMR 177 IRD-CIRAD, Centre International de Recherche-Développement sur l'Elevage en zone Subhumide (CIRDES), 01 BP 454, Bobo-Dioulasso, Burkina-Faso; 3 CNRS, Délégation Languedoc-Roussillon, Montpellier, France; 4 Centre Hospitalier, Gui de Chauliac, Montpellier, France; 5 Centre Hospitalier Universitaire Service de Parasitologie - Mycologie, Montpellier, France; 6 Department of Microbiology and Parasitology, Faculty of Medicine, Khartoum University, Khartoum, Sudan; 7 Université de la Méditerranée - INSERM UMR 906, Marseille, France; Institut Pasteur de Tunis, Tunisia

## Abstract

*Leishmania* species of the subgenus *Leishmania* and especially *L. donovani* are responsible for a large proportion of visceral leishmaniasis cases. The debate on the mode of reproduction and population structure of *Leishmania* parasites remains opened. It has been suggested that *Leishmania* parasites could alternate different modes of reproduction, more particularly clonality and frequent recombinations either between related individuals (endogamy) or between unrelated individuals (outcrossing) within strongly isolated subpopulations. To determine whether this assumption is generalized to other species, a population genetics analysis within *Leishmania donovani* complex strains was conducted within a single village. The results suggest that a mixed-mating reproduction system exists, an important heterogeneity of subsamples and the coexistence of several genetic entities in Sudanese *L. donovani*. Indeed, results showed significant genetic differentiation between the three taxa (*L. donovani*, *L. infantum* and *L. archibaldi*) and between the human or canine strains of such taxa, suggesting that there may be different imbricated transmission cycles involving either dogs or humans. Results also are in agreement with an almost strict specificity of *L. donovani* stricto sensu to human hosts. This empirical study demonstrates the complexity of population structure in the genus *Leishmania* and the need to pursue such kind of analyses at the smallest possible spatio-temporal and ecological scales.

## Author Summary

Leishmaniases are a serious public health problem, especially in developing countries, caused by *Leishmania* parasites and transmitted by sandfly bites. More information is needed on the population biology of these pathogens for diagnostic and epidemiological inquiries and for drug and vaccine elaboration. For studies dealing with the population genetics, exploring the genetic patterns of such organisms at microgeographic scales is fundamental. In this context, we made a population genetic study, based on 20 microsatellite loci, on 61 strains of *Leishmania donovani* complex collected in a Sudanese village, Babar El Fugara, during the epidemic of 1996–2000. Results showed that considering the whole sample as a single population was not adequate because of the coexistence of several genetic entities and a genetic differentiation between the human or canine strains. In addition, our findings suggested that clonality may have a strong impact on the *L. donovani* complex, unlike other *Leishmania* species. This study demonstrates the need to pursue population genetics studies in *Leishmania* species from sampling designs that control maximum possible confounding factors and to elaborate such kinds of analyses at the smallest possible spatio-temporal and ecological scales.

## Introduction

Leishmaniases are worldwide vector-borne diseases of humans and domestic animals, caused by protozoan parasites of the genus *Leishmania*. These parasitic infections are a serious public health problem, with about 350 million persons at risk and 2,357,000 new cases per year [1]. The genus *Leishmania* totals approximately 20 described species causing human infections (reviewed in [2]) with a wide variety of clinical symptoms: cutaneous, visceral, mucocutaneous, mucosal and post-kala-azar dermal (PKDL) leishmaniases. Visceral leishmaniasis is the most severe form of the disease, which can be lethal if it goes untreated. It is the most widespread leishmaniasis form, especially in India, Bangladesh, Nepal, Sudan, Ethiopia and Brazil [1], [3], [4]. In this study, we focused on human and canine samples collected in Sudan, where visceral leishmaniasis is endemic in the eastern and southern parts of the country and has claimed the lives of thousands of people [5].

Visceral leishmaniasis is mainly caused by species from the *Leishmania donovani* complex [6]. Multilocus enzyme electrophoresis [MLEE] studies generated the description of three different species in this complex: *L. donovani* in the Old World, *L. infantum* in the Old World and the New World (also named *L. chagasi* there), and *L. archibaldi* in Sudan and Ethiopia [7], [8]. In Sudan, the taxonomic status of these three species has been challenged using several different molecular markers, such as random amplified polymorphic DNA [RAPD], restriction fragment length polymorphism [RFLP] and microsatellites [9], [10]. On the basis of both sequencing and microsatellite analysis, Jamjoom et al. proposed that *Leishmania donovani* sensu lato was the only cause of visceral leishmaniasis in East Africa (the three species falling in one clade), including Sudan [11]. Lukes et al. [12], by a multifactorial genetic analysis that includes DNA sequences of protein-coding genes as well as noncoding segments, microsatellites, restriction-fragment length polymorphisms, and randomly amplified polymorphic DNAs, suggested that *Leishmania infantum* and *L. donovani* were the only recognized species of the *L. donovani* complex [12]. It was even recently suggested that the only valid name is *L. donovani* [13].

Nowadays, with the development of elaborated experimental techniques and sophisticated statistical tools, our understanding of the evolutionary processes that govern the propagation of these parasites is continuously improving. Since 1990, *Leishmania* parasites have been recognized as presenting a basic clonal mode of reproduction associated with rare recombination events [14], [15], [16]. However, recent studies based on population genetic analyses of *Leishmania* species in different environments showed strong levels of homozygosity and little amount of multilocus repeated genotypes (MLGs) [17], [18], [19], [20], [21], an observation incompatible with a strict or predominant clonal mode of reproduction [22]. More specifically, our team has proposed that *Leishmania* parasites could alternate different modes of reproduction: clonality in both vertebrate host and insect vector and recombination (recombination between related or unrelated individuals, or even interspecific recombinations) within the vector [21], [23]. The need to work within different species and at finer scales was also suggested, as the study published in Rougeron et al. showed a heterogeneity at the scale studied (country) [20], [23]. Working at finer scales indeed allows much more precise inferences to be made and a predominantly sexual signature in the genetic data. The objective of the present study was to explore such issues in another taxon, *Leishmania donovani* sensu lato within a sample collected in a single Sudanese village. We therefore analyzed the population structure of 61 *L. donovani* s.l. strains, collected in Barbar El Fugara, a village of the Atbara River region on the Sudan-Ethiopian border, at 20 polymorphic microsatellite loci. The results of this work suggest that *L. donovani* complex is a heterogeneous taxon, that dogs are not infected by the same entities as human hosts and that the different units that compose this complex are probably strongly subdivided with a significant impact of sexual recombination between related individuals. We discuss sampling strategy issues regarding further studies and insist on the need to narrow as much as possible the spatio-temporal and ecological sampling scales.

## Materials and Methods

### Study site, parasites, cultures and DNA extraction

A census of the village population was conducted by Bucheton et al. [24], making personal and clinical data available. From 1997 to 2000, 61 isolates of *Leishmania donovani* complex were collected and then cultured. We obtained the samples for this study from the “the French National Reference Center of *Leishmania*”, under the agreement of Dr. Alain Dessein.

The 61 strains from Sudan were isolated from dogs (ten strains) and humans (51 strains) and characterized using the MLEE technique by Dereure et al. [25]. Thirty-three strains were identified as *L. donovani*, 17 strains as *L. infantum* and 11 strains as *L. archibaldi* (see supplementary data Table S1). Promastigotes were cultured at 26°C by weekly subpassages in RPMI 1640 medium, buffered with 25 mM HEPES, 2 mM NaHCO_3_ and supplemented with 20% heat-inactivated fetal calf serum, 2 mM glutamine, 100 U/ml penicillin and 100 µg/mL streptomycin. Cultures were harvested by centrifugation and stored at −80°C until DNA extraction. Genomic DNA was extracted using the DNeasy Blood and Tissues Kit (Qiagen, Courtaboeuf, France), following the manufacturer's recommendations.

### Genotyping

The 20 microsatellite loci investigated (15 already published [26] and five developed in the laboratory) are listed in Supplementary data Table S1. The 61 strains (and M9702, as *L. chagasi* outgroup) under study were amplified according to the following conditions. Every 30-µL reaction mix was composed of 1 µL of each primer (10 µM), the forward being labelled, 100 ng template DNA, 0.9 µL dNTP mix (5 mM), 3 µL buffer 10× and 0.3 µL *Taq Polymerase* (Roche Diagnostics, 5 UI/µL). Amplifications were carried out in a thermal cycler using the following reaction conditions: 35 cycles of 94°C for 30 s, annealing temperature of each locus (see Table 1) for 1 min, 72°C for 1 min and a final extension step of 72°C for 10 min. The reaction products were visualized on a 1.5% agarose gel stained with EZ VISION™ DNA Dye (Amresco). Fluorescence-labelled PCR products were sized on Applied Biosystems Prism 310, with a Genescan 500 LIZ internal size standard. All 61 isolates were genotyped at all 20 loci.

**Table 1:** Description of the 20 microsatellite loci used in this study for *Leishmania donovani* complex.

Locus	Locus abbreviation	GenBank Accession no.	Allele size (bp)	Chromosome	*Ta* (°C)	*Na*	*H_s_*	*F*_IS_
*DPB1*	D1	AF182167	143–147	8	59	4	0.544	0.970
*DPB2*	D2	AF182167	235–245	8	59	6	0.526	0.688
*HG*	HG	AF170105	187–203	12	55,2	6	0.725	0.887
*Rossi1*	R1	X76394	101–115	8	59	5	0.534	0.724
*Rossi2*	R2	X76393	143–163	14	57	5	0.657	0.077
LIST7021*	L21	AF427869	216–228	36	54	3	0.423	0.884
LIST7024*	L24	AF427872	198–222	30	59	7	0.786	0.020
LIST7025*	L25	AF427873	168–212	10	56	8	0.373	0.122
LIST7026*	L26	AF427874	207–221	13	56	2	0.300	0.672
LIST7027*	L27	AF427875	185–191	26	59	4	0.501	0.967
LIST7028*	L28	AF427876	151–153	36	58	2	0.450	0.016

Locus	Locus abbreviation	GenBank Accession no.	Allele size (bp)	Chromosome	*Ta* (°C)	*Na*	*H_s_*	*F*_IS_
LIST7029*	L29	AF427877	172–176	30	56	3	0.374	0.869
LIST7030*	L30	AF427878	175–177	17	59	2	0.341	0.904
LIST7031*	L31	AF427879	164–208	10	54	3	0.290	0.208
LIST7033*	L33	AF427881	176–206	25	58	6	0.611	0.866
LIST7034*	L34	AF427882	143–171	12	54	5	0.254	0.677
LIST7035*	L35	AF427883	185–199	23	56	3	0.376	0.956
LIST7037*	L37	AF427885	171–201	21	58	5	0.562	0.067
LIST7038*	L38	AF427886	128–146	26	56	3	0.326	0.598
LIST7039*	L39	AF427887	190–192	30	58	3	0.537	0.847
Mean ± standard error				…		4.25±1.74	0.475±0.15	0.584±0.3

The following parameters are described: name, abbreviation, Genebank accession number, allele size (bp), chromosome localization, thermocycling conditions (annealing temperature, *T_a_*), genetic variation (alleles number), *Na*; average estimate within-sample gene diversity *H*_S_, and deviation from panmixia measured as *F*_IS_. The loci noted by ‘*’were developed by Jamjoom *et al.* [26].

### Statistical analysis

Data were processed through Create V 1.1 [27] to convert the data for different usage. We mainly analysed data with Fstat Version 2.9.3.2 software (Goudet 2002, updated from Goudet [28]), which computes estimates and tests the significance of the following population genetics parameters. Genetic polymorphism was measured by the number of alleles per locus (*N*_a_) and by Nei's unbiased estimate of genetic diversity within subsamples *H_s_* [29]. We estimated Wright's *F* statistics [30] with Weir and Cockerham's method [31]: *F*_IS_ measures the relative inbreeding of individuals due to the local non-random union of gametes in each subpopulation, and *F*_ST_ measures the relative inbreeding in subpopulations attributable to the subdivision of the total population into subpopulation of limited size. *F*_ST_ thus also measures genetic differentiation between subpopulations. *F*_IS_ ranges between −1 and 1: a negative value corresponds to an excess of heterozygotes, a positive value to heterozygote deficiency; 0 is expected under panmixia. The significance of the departure from 0 was tested by 10,000 randomisations of alleles within subpopulations (to test random mating) and individuals across subsamples (for differentiation). The statistic used for random mating (Hardy-Weinberg Equilibrium) testing was simply Weir and Cockerham's estimator *f* (*F*_IS_ and *F*_ST_). For the genetic differentiation test, we used the log likelihood ratio *G*-based test of Goudet et al. [32] summed over all loci. Confidence intervals were estimated by bootstrapping over loci or jack-knifing over populations with Fstat as described in De Meeûs et al [33].

Genetic diversity, as measured by Nei's *H_s_*, can lower the maximum possible value for *F*_ST_. According to classical formulation (e.g. [34] *F*_ST_ = (*Q*_S_−*Q*_T_)/(1−*Q*_T_), where *Q*_S_ is the probability to sample twice the same allele in a subpopulation and *Q*_T_ is the probability to sample twice the same allele in different subpopulations. If a population was totally subdivided, then the probability to sample twice the same allele in two different subpopulations should be null and thus *F*_ST_ should be equal to the probability to sample twice the same allele in a subpopulation *Q*_S_. *H_s_* being the probability to sample two alleles that are different hence *Q*_S_ = 1−*H_s_*. The maximum possible value for *F*_ST_ in a sample with a given *H_s_* can thus be estimated as 1−*H_s_* and a corrected version of *F*_ST_ as *F*_ST_′ = *F*_ST_/(1−*H_s_*) [33], [35].

Data were heterogeneous regarding *Leishmania* species (as recognized by MLEE typing), year of sampling and host species. To assess the possible contribution of these factors to genetic partitioning (Wahlund effect), we compared *F*_IS_ obtained with four different sampling strategies. The first sampling strategy considered each *Leishmania* species-year of sampling–host species combinations as different subsamples (14 subsamples, “All separated” strategy). The second strategy ignored the *Leishmania* species distinction (six subsamples, “Species fused” strategy). The third strategy ignored the year of sampling (six subsamples, “Years fused” strategy) and the fourth one ignored the host species (10 subsamples, “Hosts fused” strategy). For significant difference testing, we undertook planned paired Wilcoxon signed rank tests between “All separated” and each of the other three strategies ordered as above with sequential Bonferroni correction (multiplying the *P*-values by 3, 2 and 1, respectively). Unilateral (“All separated” has a smaller *F*_IS_ than the other three strategies) Wilcoxon signed rank tests were undertaken under R [36]. Differentiation between the relevant units controlled for the other factors were then undertaken with paired subsample differentiation tests (*F*_ST_ estimation and *G*-based randomisation test). When two values were obtained for the same type of differentiation (e.g. differentiation between *L. archibaldi* and *L. infantum* in 1997 and 1998), these values were combined with an unweighted mean for *F*_ST_ (e.g. over years) and Stouffer's *Z* test (Whitlock, 2005) for *P*-values as recommended [37].

Linkage disequilibrium between pairs of loci (non-random association of alleles at different loci) was assessed with a randomisation test (genotypes at two loci are associated at random a number of times) using Fstat software Version 2.9.3.2 software (Goudet 2002, updated from Goudet [28]). The statistic used was the log likelihood ratio *G* summed over all subpopulations, known to be more powerful than other combinatory procedures [37]. Because there are as many tests as locus pairs tested (here 15×14/2 = 190), we expected 0.05×190∼9.5 significant tests under the null hypothesis of no linkage disequilibrium at significance level *α* = 0.05. Thus we used the unilateral (“greater”) exact binomial test to check if there was significantly more than 5% significant tests in the 190 tests series under R [36].

The BAPS version 5.1 software identifies a hidden structure within populations (admixture) through a Bayesian analysis [38]. This software was used to detect possible Wahlund effects and has been successfully applied to other parasites [21], [39], [40]. The BAPS software uses stochastic optimization to infer the posterior mode of genetic structure. To obtain the best distribution of the entire population, we ran the program 50 times in order to obtain the right number of clusters. The same approach has been applied within *L. donovani* 1997, *L. donovani* 1998 and *L. infantum* from humans for which enough individuals were available. Each of the three samples was submitted to a clustering exploration by BAPS with a maximum number of clusters set (19, 13 and 12, respectively, these values corresponding to the number of individuals in each sample). *F*_IS_ was recalculated in each best distribution identified by BAPS and noted *F*_IS___C_. Then, for the three samples corresponding to the three species of *Leishmania*, the *F*_IS___C_ was compared with the initial *F*_IS_ using a unilateral Wilcoxon signed-rank test for paired data (with the software R), the pairing units being the 20 loci. If *F*_IS___C_ is significantly lower than *F*_IS_, it is probable that the initial subsamples were composed of several genetically distinct entities (e.g. geographical microstructure or subpopulations).

Since we got the data's prevalence from Dereure et al. study [25], the prevalences were compared for each *Leishmania* species between humans and dogs (50 human strains and 20 dog strains), and the significance was tested using an exact Fisher test under the software R [36].

A Neighbor-Joining (NJ) tree [41] was constructed out of a Cavalli-Sforza and Edwards genetic distance matrix [42]. The robustness of tree topology was obtained by bootstrap resampling of loci, with 500 replications per set. We used PHYLIP software (version 3.5c; J. Felsenstein, Department of Genetics, University of Washington, Seattle, 1993) and the tree was edited using TreeDyn software [43].

Simulations where also handled with Easypop 2.0.1 (Balloux 2006, updated from Balloux 2001 [44]) to find possible sets of parameters fitting our observations.

### Ethical statement

The approval for human strain study was obtained by both the federal and state Ministries of Health and by the Faculty of Medicine of Khartoum. Approval of the project to be performed was also approved for each field visit by the village committee, which included elected delegates from all ethnic groups and as well elected citizens. Since an important proportion of the population in Barbar El Fugara was illetrate, oral informed consent was obtained after the aim of the study was explained to study participants in their own language by a translator. For child participants, oral consent was obtained from their parents. The verbal consent was also obtained in the presence of the ethic group leader, who eventually provide more explanations if required. After verbal informed consent obtained from the patient, the clinician recorded it on a written form.

## Results

We obtained clear electrophoregrams for all genotypes at all 20 loci investigated, with only one or two alleles per strain at each locus, which excludes events of aneuploidy (for which we would have also expected individuals with no alleles, three or four alleles). The genotypes obtained are presented in supplemental Table S1. The data showed a low level of genetic diversity, with an average number of alleles per locus of 4.25±1.74, ranging from 2 (LIST7026, LIST7028 and LIST7030) to 8 (LIST7025) and a mean genetic diversity *H*_S_ = 0.475±0.148 (Table 1).

### Phylogenic analysis and genetic differentiation

**Figure 1:**
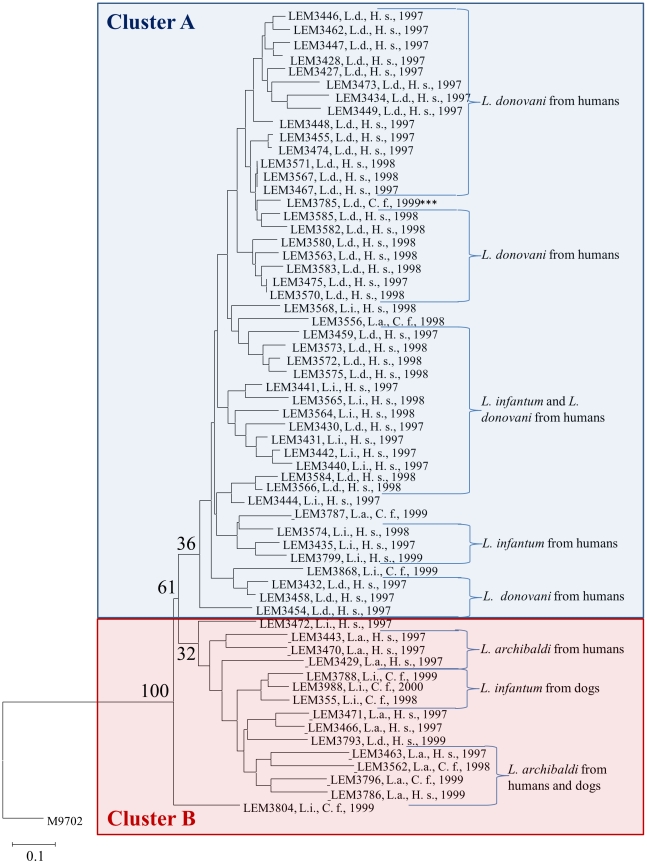
Genetic distance relationships among 61 strains of the *Leishmania donovani* complex in Sudan. Neighbor-Joining tree depicting genetic distance relationships based on Cavalli-Sforza's chord distances among 61 strains of the *Leishmania donovani* complex based on 20 polymorphic microsatellite loci. *Leishmania chagasi* M9702 reference strain was used as outgroup. Values on the nodes represent the percentage of bootstrap replicates over loci (*n* = 500). Samples from the species *L. archibaldi*, *L. donovani* and *L. infantum* are noted “L. a.”, “L. d.”, and “L. i.” respectively. Hosts are noted “H. s.” for *Homo sapiens* and “C. f.” for *Canis familiaris*. The majority of *L. archibaldi* strains are grouped in cluster B. Cluster A contains almost all the *L. donovani* strains. *L. infantum* strains are distributed in the two clusters. The single *L. donovani* strain (LEM3785) genotyped from a dog is noted “***”.

The dendrogram, based on 20 polymorphic microsatellite loci, represented in Figure 1 underlined two main clusters. Cluster A (36% bootstraps) regroups strains from *L. infantum* and *L. donovani*. Cluster B (sustained by a bootstrap of 32%) corresponded to *L. archibaldi* taxon and three *L. infantum* from dogs. It has to be noticed that other studies have observed, using microsatellite method, small bootstrap for large clusters and important bootstrap values only for small clusters for *L. braziliensis* [45] and *L. infantum* [46].

**Figure 2:**
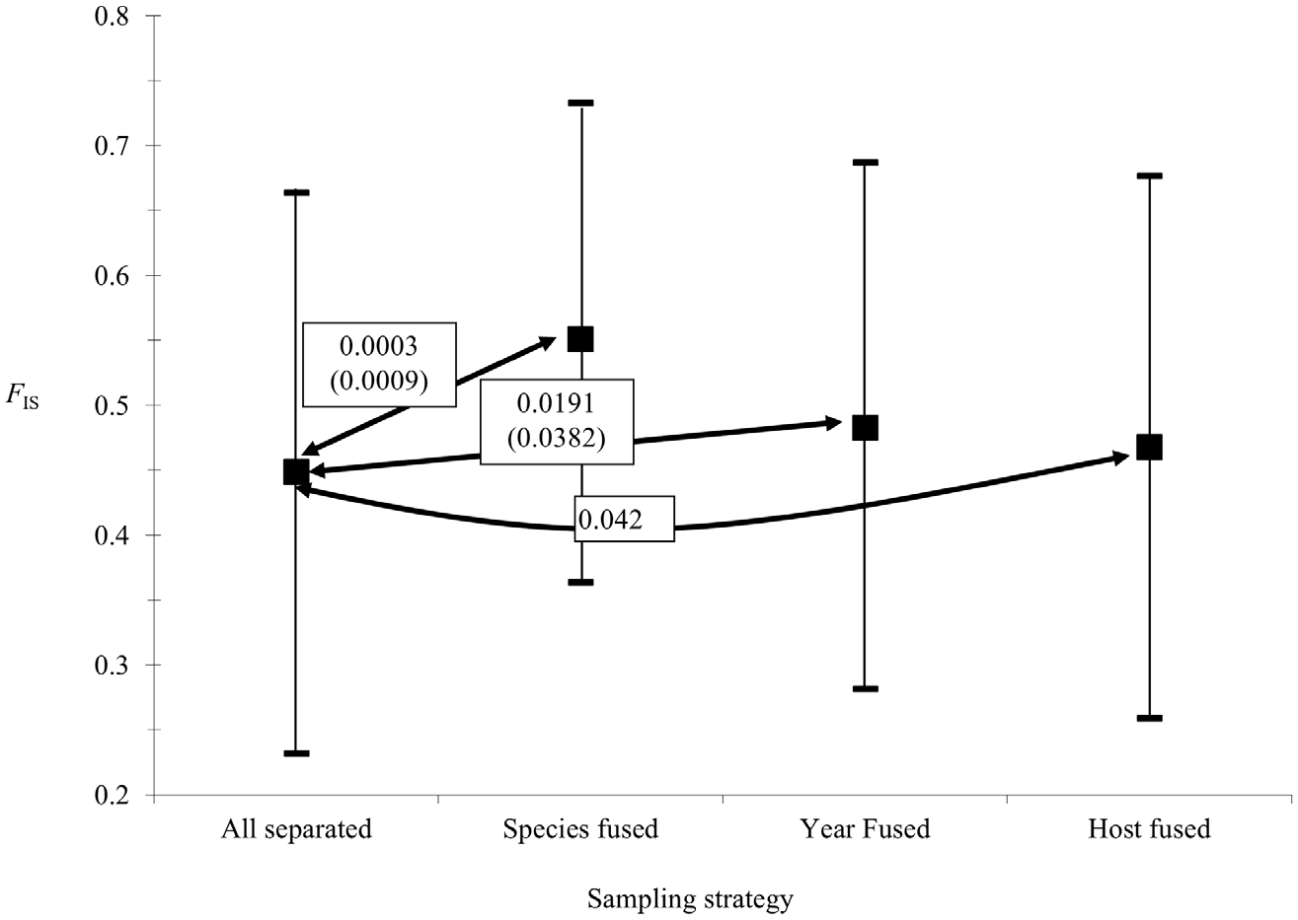
*F*_IS_ estimations between four sampling strategies. Comparison of *F*_IS_ estimated between the “All separated” strategy and the other three sampling strategies as defined in the text. Wilcoxon signed rank test *P*-values between pairs of strategies, as indicated by double arrows, are provided with the corresponding sequential Bonferroni corrected value in brackets. The 95% confidence intervals were obtained by bootstrapping over loci.

*F*_IS_ comparisons between “All separated” strategy and the three others gave significant differences, as illustrated in Figure 2, meaning each factor, *Leishmania* species, year of sampling and host species in order of importance, displays a significant signature on the apportioning of genetic information. Consequently, each *Leishmania* species of each year and each host species must be considered as separate subsamples. It has to be noticed that the significant results we obtained cannot come from an insufficient number of samples. Indeed, the significant differences evidenced are statistically valid and ignoring it might lead to overlook important ecological processes currently involved in the population biology of these *Leishmania* “lineages”. Moreover, these differentiations were confirmed by paired subsample differentiation tests, as indicated in Table 2. All *Leishmania* species are genetically different. Species differentiation seems very pronounced between *L. donovani* and *L. archibaldi* (*F*_ST_′∼0.767) and smaller for the two other pairs (*F*_ST_′∼0.2–0.3) (Table 2). Temporal differentiation seems only to affect *L. donovani* in humans. Considering the host origin, a weak and marginally non-significant differentiation is found between human and dog strains for *L. archibaldi*, while a strong differentiation seems to affect *L. infantum* strains between the two host species (Table 2).

**Table 2:** Differentiation measures (*F*_ST_) and testing (*P*-value) between different *Leishmania donovani* sl strains.

Comparison	Sub-samples	*F*_ST_	*P*-value	*H_s_*	*F*_ST_′
	*L. archibaldi vs L. donovani* (1997, human)	0.4758	0.0001	0.3800	0.7674
	*L. archibaldi vs L. infantum* (1997, human)	0.3738	0.0013	0.4050	0.6282
Species	*L. archibaldi vs L. infantum* (1999, human)	−0.0464	0.3970	I	I
	Mean (*L. archibaldi vs L. infantum*, human)	0.1637	0.0103	0.5205	0.3414
	*L. donovani vs L. infantum* (1997, human)	0.1738	0.0001	0.3240	0.2571
	*L. donovani vs L. infantum* (1998, human)	0.1386	0.0015	0.3210	0.2041
	Mean (*L. donovani vs L. infantum*, human)	0.1562	0.0001	0.3225	0.2306
	1997 *vs* 1998 (*L. donovani*, human)	0.1017	0.0001	0.2780	0.1409
Years	1997 *vs* 1998 (*L. infantum*, human)	−0.0495	0.7725	I	I
	1998 *vs* 1999 (*L. archibaldi*, dog)	−0.1943	0.6624	I	I
Hosts	In *L. archibaldi*, ignoring years	0.0495	0.0708	0.5850	0.1193
	In *L. infantum*, ignoring years	0.2872	0.0009	0.4210	0.4960

I: Irrelevant.

These estimations have been calculated according to the species (as defined by MLEE), year of sampling and host species and controlling for the other factors (only possible on some occasions). As year of sampling did not seem to greatly influence differentiation in *L. archibaldi* and *L. infantum*, years were ignored in host species comparisons in these two species (no possible tests otherwise). The results from comparable analyses were combined with an unweighted mean (for mean *F*_ST_) and Stouffer's *Z* test [62] (for *P*-value). *H_s_* and Standardised values for *F*_ST_, *F*_ST_′ = *F*_ST_/(1−*H_s_*) are also given when appropriate.

Clinical forms (visceral versus PKDL in humans, see Supplementary Table S1) could only be compared for *L. donovani* in 1997 and 1998 where no differentiation could be evidenced (*F*_ST_∼0, *P*-value>0.4 in both cases). Consequently, clinical forms were not considered further in our analyses.

### Prevalence comparisons

The data's prevalence from Dereure et al. [25] was compared for each *Leishmania* species between humans and dogs (50 human strains and 20 dog strains). The results, presented in Table 3, show that *L. donovani* is clearly found in humans rather than in dogs (*P*-value = 0.001), that *L. infantum* displays a tendency to infect dogs more often (*P*-value = 0.04), while the difference is not significant for *L. archibaldi* (*P*-value = 0.2). If Bonferroni adjusted, only *L. donovani* test stays significant (*P*-value = 0.003).

**Table 3:** Comparison between prevalence on humans and dogs for the different species of *Leishmania*.

Parasite	Host	Infected	Non infected	*P*-value
*L. archibaldi*	Humans	7	45	0.169
	Dogs	6	14	
*L. donovani*	Humans	33	19	0.001
	Dogs	4	16	
*L. infantum*	Humans	12	40	0.044
	Dogs	10	10	

*P*-values correspond to the results obtained with the Fisher's exact test [25].

### Linkage disequilibrium study

This analysis was undertaken over all the data but considering each *Leishmania* species, year of sampling and host species combination as a distinct subsample. This provided 19 locus pairs out of 190 tests in significant linkage. This is far above the 5% expected under the null hypothesis (*P*-value = 0.0001). These significant tests involved 18 of the 20 loci. Within each *Leishmania* species, small subsample sizes limited the power of the test. For *L. archibaldi* (very small subsamples of four and seven individuals in dogs and human hosts respectively) only five tests out of 190 were significant (*P*-value = 1). In *L. donovani* 22 tests were significant (*P*-value = 0.0003) and in *L. infantum* 19 tests were significant (*P*-value = 0.0034). There is thus a global linkage at a genome-wide scale in the three *Leishmania* species populations.

### Genetic diversity and heterozygote deficiency within *Leishmania* species

**Figure 3:**
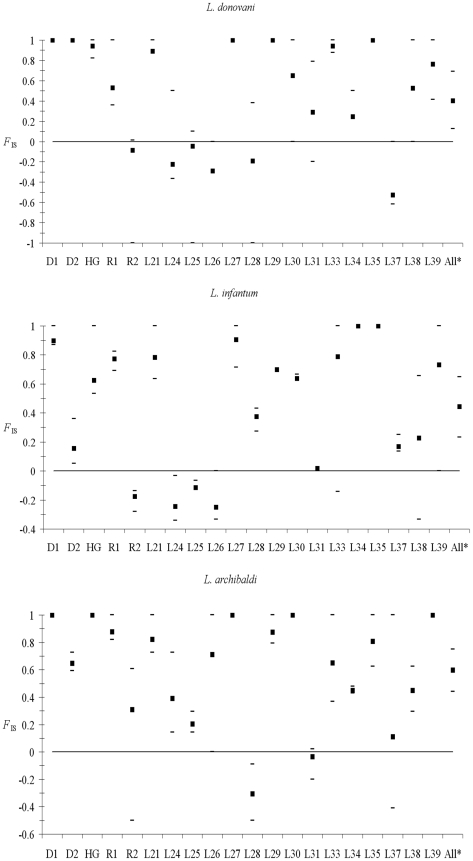
*F*_IS_ variation across loci and mean value for the three *Leishmania* species. The confidence intervals are the values obtained for dogs and humans for *L. archibaldi* and *L. infantum* and are minimum and maximum values obtained in 1997, 1998 or 1999 for *L. donovani*, except for *F*_IS_ over all loci (All*) where confidence intervals (CI) are the 95% CI obtained after bootstrap over the loci.

**Figure 4:**
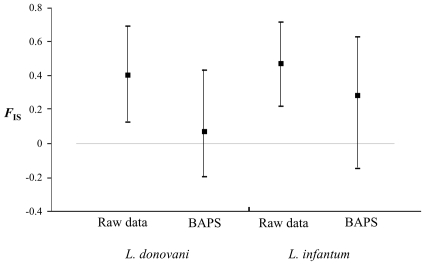
*F*_IS_ for *L. donovani* and *L. infantum* strains in the entire population and within subdivisions. These subdivisions have been identified by the software BAPS. The 95% confidence intervals were obtained by bootstrapping over loci. The decrease of *F*_IS_ in the subdivisions suggests a Wahlund effect.

For each *Leishmania* species, a global and highly significant heterozygote deficit, highly variable across loci, was observed (Figure 3). These heterozygote deficits significantly decrease (*P*-values<0.005) in the best partitions found by BAPS for the two species for which such analyses could be done (*L. donovani* and *L. infantum*) (Table 4 and Figure 4). Simulations, undertaken using the software EasyPop, provided patterns convergent with the pattern observed for some parameter sets only for very high clonal rates (minimum *c* = 0.99) and strong Wahlund effects (pooling one representative of each strongly isolated subpopulation into one subsample). Nevertheless, in each of these simulations, fairly numerous multilocus genotypes (MLGs) appeared, in contrast to the real data, where on the whole data set only two MLGs (2 observations of two samples presented the same multilocus genotypes) were observed. Consequently, something else is occurring. Finally, using the NJ Tree pattern of Figure 1, keeping only *L. donovani* strains belonging to most homogeneous clusters (no leaf longer than 0.1, see Figure 1) and subdividing it into subclusters belonging to the same year indeed produced lower *F*_IS_∼0.27, but still with a very strong variance across loci (ranging from −0.1 to 0.7), no significant linkage disequilibrium and a reasonable proportion of MLGs (one repeated twice and a second repeated three times) but very small subsample sizes. It has to be noticed, that the global same topology of the NJ tree using Cavali Sforza distances has been obtained using shared allele distances, and also the Minimum Evolution tree using either Cavali Sforza and shared allele distances.

**Table 4:** Description of the clusters identified using the software BAPS.

	Subsamples	Individuals per cluster	Number of clusters	*P*
*L. donovani*	1997	10	1	
		2	3	0.986
		1	2	
	1998	4	2	
		3	1	0.996
		2	1	
*L. infantum*	Human hosts	6	1	
		3	1	0.813
		2	1	
		1	1	

Number of clusters, their size and probability of best partition (*P*) during BAPS analyses of *L. donovani* samples in 1997 and 1998 and of *L. infantum* from human hosts (other subsamples were too small).

## Discussion

Despite the latest studies in this area, the debate on population structure and *Leishmania* reproductive mode is far from being settled and therefore deserves further investigation. Recent publications on different *Leishmania* species and in different environments seriously challenge the view that the species of the genus should display a predominantly clonal genetic signature because of important homozygosity levels and rarity of MLGs [17], [18], [19], [20], [21]. As suggested for *L. braziliensis* [21], these parasites could alternate different modes of reproduction: clonality in both vertebrate host and insect vector and sexual recombination (similar to other kinetoplastid parasites, such as *Trypanosoma brucei* s.l. [47], or other Trypanosomatidae such as Crithidia bombi [48]) between genetically related cells (endogamy) resulting in high levels of inbreeding. Most of these studies also revealed strong heterogeneities within *Leishmania* subsamples that probably results from Wahlund effects (mixture of differentiated true populations), because strains were collected at too large spatial and/or temporal scales. To prevent such possible biases, we selected a sample of *L. donovani*, collected at a village scale, reducing the risk of hidden substructuring.

In this Sudanese village, the validity of the distinction between *L. donovani* sensu stricto, *L. archibaldi* and *L. infantum*, be it a true species, a subspecies or any other taxonomic level, is supported by our results, in contradiction with recent papers [9], [11], [13], [49]. As shown here, ignoring such delimitations dangerously biases genetic data interpretation. It remains that taxonomic distinction based on isoenzymes does not seem very clear as can be seen from Figure 1 and it would be worth trying other kind of markers as MultiLocus Sequencing Typing or MultiLocus Sequencing Analysis [50] to clarify this issue.

Another significant subdivision arose between dogs and human hosts, particularly regarding *L. infantum* and to a much lesser extent *L. archibaldi*. Gene flow (gene flow) appears much reduced between dogs and human hosts for *L. infantum* and two different kinds of cycles must be present here, involving probably different vector's species and reservoirs. For *L. archibaldi* the difference is much less obvious but may be as a result of modest sampling sizes. In *L. donovani*, the greater specificity of strains to human (Table 3) and the resulting reduced number of strains found in dog did not allow for such testing. Nevertheless, the single *L. donovani* strain (LEM3785) genotyped from a dog did not show any originality as regard to its human counterparts (Figure 1). This apparent strong specificity for human hosts would mean, at least for *L. donovani*, that dogs are not a significant reservoir for these pathogens, in agreement with the anthroponotic feature of this species.

Time also appeared as a significant subdividing factor but only for *L. donovani*. The simplest interpretation being that, as patient once diagnosed are treated, a drop in subpopulation size may occur in the following year, thus leading to a genetic differentiation as a result of a bottleneck or of the replacement of empty places by other strains. Genetic diversities being not significantly different between 1998 and 1999 (Wilcoxon signed rank test, *P*-value = 0.27), the second hypothesis appears more likely.

Failing to consider all the above factors as relevant resulted in a very odd *F*_IS_ distribution as illustrated by Supplementary Figure S1.

Our data, and especially the NJTree approach, also suggest that hybridization between the different taxa is not impossible, though rare enough to prevent homogenization, but frequent enough to enhance heterogeneity within each cluster that could be defined.

An interesting point to notice is the absence of genetic differentiation obtained between *L. donovani* clinical forms (visceral leishmaniasis and PKDL, *F*_ST_∼0, *P*-value>0 in 1997 and 1998). Indeed, this result could suggest that the development of PKDL in treated patients is more likely link to host's factors than to parasite's factors. This potential association between PKDL and host has already been suggested by Blackwell J.M.'s team. Indeed, results of this study proposed a genetic association between the polymorphism at IFNGR1 and the susceptibility of patients after treatments to PKDL (and not to visceral leishmaniasis) [51].

Regarding the reproductive strategy and population structure of these parasites, further studies should focus on the effect of individual hosts to detail the respective contribution of population differentiation as well as clonal, endogamic and outcrossing modes of reproduction in the genotypic distribution of these parasites. Nonetheless, clonality does not totally explain the strong variance across *F*_IS_ loci, that displayed a much wider range than what was observed for the much more homozygous *L. braziliensis* [21]. Our simulation approach suggested that obtaining the *F*_IS_ and its variance across loci with very few MLGs, as in the real data, was impossible to achieve. The existence of a strongly structured hierarchical meta-population, with for instance the individual hosts playing the role of micro-populations for the parasites, in combination with occasional gene flow between different genetically distant entities (species hybridizations) and/or different cycle types (zoonotic vs. anthroponotic), could explain the pattern observed on our microsatellite loci. However, as previously said, this requires further investigation. We cannot exclude the possible disturbing role played by gene conversion known to occur in *Leishmania* [52] though we do not favour much such a hypothesis. If gene conversion is a genome wide process in *Leishmania* (genomic conversion) we would have expected a much more homogeneous homozygosity across loci than what was observed. Some loci are indeed almost always homozygous while some others display substantial amounts of heterozygosity (Figure 3). If gene conversion is site specific, we would expect it to preferentially affect coding sequences and its surrounding more than non coding zones. A glance at the localisation in the chromosome of markers did not suggest that microsatellite markers situated closer to coding sequences were more prone to display positive *F*_IS_ than the other microsatellites. Moreover, even if the correlation between species is good, it can be seen that it is not perfect and that some loci with *F*_IS_∼1 in one species can display a fairly lower *F*_IS_ in another. This does not strongly support the site specific DNA conversion hypothesis. But here again, further studies would be worth being undertaken on that issue.

Null alleles are often encountered in population genetics studies. They may be frequent in allozymes [53], [54] and in DNA markers such as microsatellites [55], [56], [57]. In our data, no blank has ever been observed in the genotypes (no missing data, i.e. all individuals were amplified at all loci), which, given the high homozygosity encountered (increased probability of blank homozygotes), makes the null allele explanation very unlikely.

Rarity of MLGs, variable but globally positive *F*_IS_ and strong heterogeneity within subsamples seem to be the rule for *L. donovani* as such a pattern was already reported in Eastern Africa [19]. Such results suggest the existence of strongly differentiated hidden entities. A different pattern was found in *L. donovani* from the Indian subcontinent [58] where all loci appeared weakly polymorphic, dominated by a single MLG with a few variants at one locus and, in spatially and temporally homogeneous subsamples no deviation from panmixia. Just as if this subcontinent had been colonised by one of the entities we are dealing with Africa.

The village Babar El Fugara is characterized by an epidemic context, with the occurrence of several epidemic episodes. The genetic diversity revealed by our results is not due to the arrival of a new variant but more likely was already present. Indeed, during this epidemic, all the population have been exposed to the disease and only ¼ develop visceral leishmaniases. This observation means that the majority of the population is probably asymptomatic and constitute a reservoir for the transmission [24]. In this context, this suggested the need to pursue research in order to identify which reservoir could be involved in the maintenance of the diversity and the transmission cycles (vectors or mammal reservoirs).

To conclude on this population genetics study within the *L. donovani* complex, it clearly appears that considering the whole sample as a single population was not adequate. In addition, our findings suggested that clonality may have a stronger impact on the *L. donovani* complex than on *L. braziliensis*. It also suggested that exploring the possible strong impact of the host individual (sandfly or mammal hosts) was worth trying and indeed represents a too often neglected factor in *Leishmania* population studies in particular and in pathogenic microbes in general [22], [33], [59], [60], [61]. These results demonstrate the need to pursue population genetics studies in *Leishmania* species from sampling designs that control maximum possible confounding factors. These parasites indeed seem to be subdivided at very narrow spatio-temporal and ecological (host) scales.

## Supporting Information

Figure S1 ***F*** **_IS_ for each of the loci in the entire population of *L. donovani* complex.** There is a large heterozygote deficiency at each locus.
(TIF)

Checklist S1 **STROBE checklist.** Checklist of items included in this population genetic study.
(PDF)

Table S1 **Description of data set and microsatellite genotypes.** Each sample is detailed by sample code, species attribution by MLEE [25], host, clinical forms (VL for Visceral Leishmaniases and PKDL for PostKala azar Dermatite Leishmaniases) and year of collection, and microsatellite genotypes obtained at each locus.
(XLS)
