# Supplementary figures and images for: Multifaceted Population Structure and Reproductive Strategy in Leishmania donovani Complex in One Sudanese Village
Source: PLoS Negl Trop Dis. 2011 Dec 20;5(12):e1448. doi: 10.1371/journal.pntd.0001448 (PMC3243727; doi:10.1371/journal.pntd.0001448)

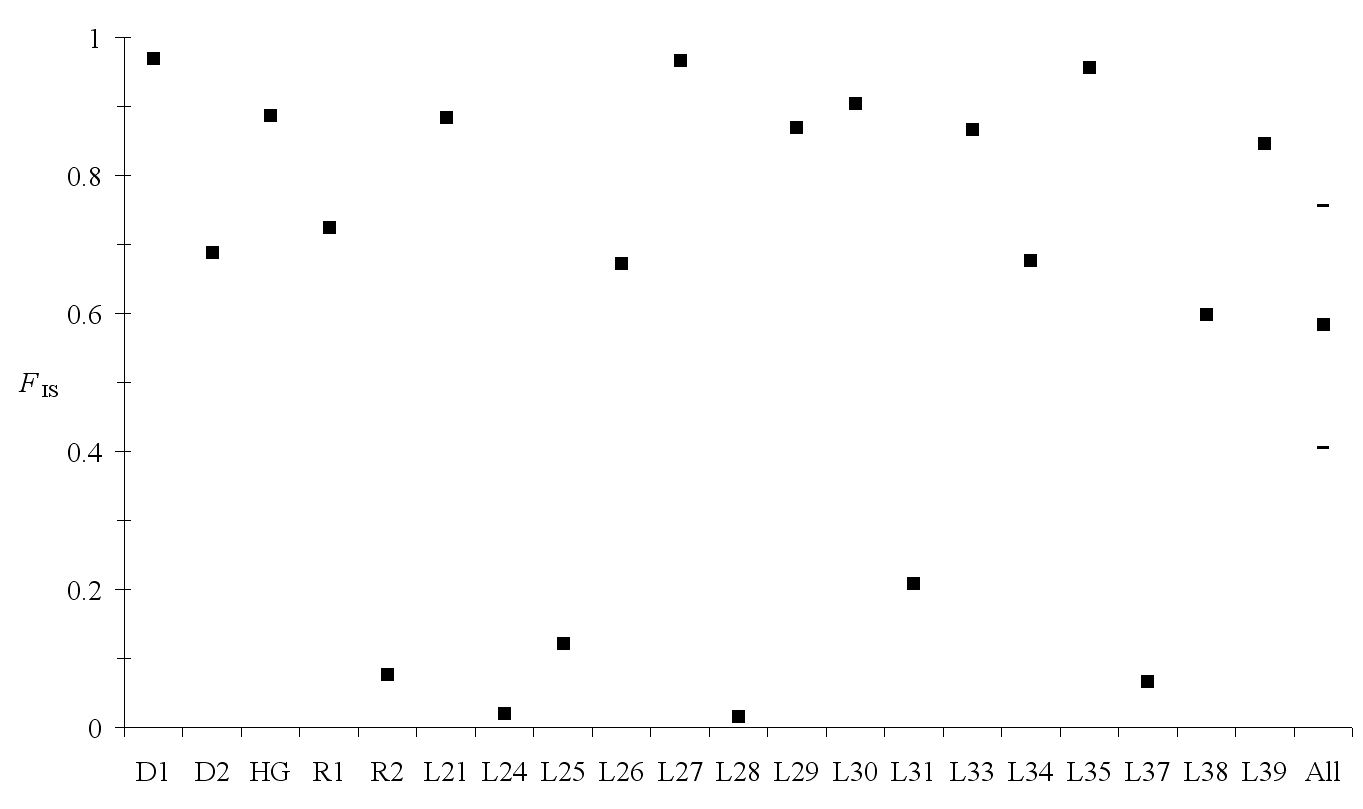

Supplement: Figure S1 — F IS for each of the loci in the entire population of L. donovani complex. There is a large heterozygote deficiency at each locus. (TIF) [file pntd.0001448.s001.tif]
